# Latent Structure and Profiles of Emotion Regulation: Reappraisal and Suppression Patterns with the Polish Version of the Emotion Regulation Questionnaire

**DOI:** 10.3390/jcm14020587

**Published:** 2025-01-17

**Authors:** Paweł Larionow, Karolina Mudło-Głagolska, David A. Preece

**Affiliations:** 1 Faculty of Psychology, Kazimierz Wielki University, 85-064 Bydgoszcz, Poland; 2 School of Population Health, Curtin University, Perth, WA 6102, Australia; david.preece@curtin.edu.au; 3 School of Psychological Science, The University of Western Australia, Perth, WA 6009, Australia; 4 Department of Psychology, Stanford University, Stanford, CA 94305, USA

**Keywords:** alexithymia, anxiety, cognitive reappraisal, depression, Emotion Regulation Questionnaire, expressive suppression, latent profile analysis, psychometric properties, psychopathology, well-being

## Abstract

**Background/Objectives**: The Emotion Regulation Questionnaire (ERQ) is a 10-item self-report measure of two emotion regulation strategies, cognitive reappraisal (CR) and expressive suppression (ES). This study aimed to (1) examine the latent structure of the Polish version of the ERQ, and (2) use it to explore different profiles of emotion regulation strategy use and their links with mental health outcomes. **Methods**: Our sample was 1197 Polish-speaking adults from the general community in Poland. **Results**: A factor analysis showed that the ERQ had strong factorial validity, with an intended two-factor structure (CR and ES factors) that was invariant across gender, age, and education categories, as well as across different levels of psychopathology symptoms and alexithymia. Our latent profile analysis extracted four emotion regulation profiles (subtypes): a Mainly Reappraisal profile (high CR, low ES), a Mainly Suppression profile (low CR with high ES), a Generally Low Regulation profile (low CR, low ES), and a Generally High Regulation profile (high CR, high ES). People with the Mainly Reappraisal profile had the best mental health outcomes, whereas people with the Mainly Suppression profile had the poorest mental health outcomes. **Conclusions**: Conceptually, these findings support the *process model of emotion regulation*, illustrating the differential affective outcomes of various emotion regulation strategies. Our results highlight the importance of considering individual differences in strategy use patterns, including combinations of strategies within an emotion regulation profile. The Polish version of the ERQ appears to be a robust measure of these key emotion regulation processes across a variety of demographic groups. To facilitate its use, including score interpretations in clinical practice, we present Polish percentile rank norms for the ERQ.

## 1. Introduction

Emotion regulation refers to the processes by which people modify their experience and expression of emotions [1]. Strong emotion regulation is a significant contributing factor to the maintenance of well-being, whereas poor emotion regulation abilities contribute to the development of a wide range of psychopathologies [2,3,4,5].

### 1.1. The Process Model of Emotion Regulation

The process model of emotion regulation is one of the most widely used and well-evidenced theoretical frameworks of emotion regulation [1,6]. This model describes five broad families of emotion regulation strategies, including (1) situation selection (changing the emotion-eliciting situations one is exposed to), (2) situation modification (changing the nature of the emotion-eliciting situations one is in), (3) attentional deployment (changing what aspects of the emotion-eliciting situations are focused on), (4) cognitive change (changing the way one is thinking about emotion-eliciting situations), and (5) response modulation (changing the experience or expression of the emotion once it is more fully developed).

### 1.2. Cognitive Reappraisal and Expressive Suppression and Their Links to Mental Health

Amongst the most studied emotion regulation strategies are cognitive reappraisal (CR) and expressive suppression (ES) [7]. Cognitive reappraisal belongs to the cognitive change family of strategies, and refers to looking at a situation from different points of view to change its emotional influence. This strategy is considered as generally adaptive, with its habitual use usually linked to higher well-being and lower ill-being (e.g., [1,8,9,10]).

In contrast, expressive suppression belongs to the family of response modulation strategies, and refers to suppressing the behavioral expression of the emotion (i.e., not showing emotions through facial expressions or voice intonation). Expressive suppression is generally considered as a maladaptive strategy, with its habitual use usually linked to lower well-being and higher ill-being (e.g., [1,8,9,10]).

### 1.3. Latent Structure of the Emotion Regulation Questionnaire (ERQ)

The examination of these strategies has been enabled by psychometric measures like the Emotion Regulation Questionnaire (ERQ) [9], a 10-item self-report measure of how frequently people use CR or ES, as well as its 6-item short form (the ERQ-S) [11]. The psychometric properties of the ERQ have been examined across a range of countries and sample types (e.g., [10,12,13,14,15,16,17,18]).

Factor analytic studies to date support a two-factor structure, comprising CR and ES factors, thus supporting the separability of these strategies in a latent modeling framework (e.g., [10,12,13,14,15,16,17,18]). The two factors tend to be largely uncorrelated, highlighting their separability as different regulation strategy approaches. This structure has been found to be invariant across various demographic groups, and both factors appear to have good reliability, for example, with Cronbach’s alpha and McDonald’s omega coefficients usually over 0.75 (e.g., [17]).

### 1.4. The Emotion Regulation Questionnaire (ERQ) in the Polish Context

Most work with the ERQ has so far been performed in English-speaking samples (e.g., [9]) and there is limited data on the ERQ in the Polish context [19,20,21]. As such, there is a pressing need for the psychometric validation of a Polish version of the ERQ.

Previously, a Polish form of the 6-item ERQ-S was translated and validated by Larionow et al. [19], showing good psychometric performance (e.g., a theoretically congruent two-factor structure, high reliability, and coherent correlations with other relevant measures), but to date, there are no published validation studies of the 10-item ERQ in Polish. Given the high popularity of the full ERQ in the wider affective science field, this is an important gap to address.

Thus, one of our aims here was to introduce and validate a Polish version of the full 10-item ERQ. In so doing, this helps to further establish the cross-cultural structure and applicability of emotion regulation.

### 1.5. Profiles of Emotion Regulation and Mental Health Outcomes

In terms of examining patterns of emotion regulation with valid assessment tools, there are two main methodological approaches: a variable-centered and a person-centered approach (see Van Eck et al. [22]). In this field, the variable-centered approach and its key analyses (e.g., correlational and regression analyses) have been the most common methodologies, whereas the person-centered approach (e.g., latent profile or cluster analyses) has been relatively rarely used (see Van Eck et al. [22], Guérin-Marion et al. [23], Pinto et al. [24], Zeng et al. [25]). Whilst both have important utility, one advantage of including the person-centered approach is it allows the exploration of the possibility that the relationship between variables may not be the same for all participants. With this approach, it is possible to distinguish subtypes of people that have unique profiles across a range of variables of interest, examining how common such subtypes are. In the field of emotion regulation, this may be helpful in assessing how combinations of emotion regulation strategies fit within a profile, and better personalizing treatment targets to the unique needs of individuals [24,26].

To date, there are few studies on emotion regulation that have used a latent profile analysis to examine profiles of emotion regulation strategy use. Two example studies are by Zeng et al. [25] and Eftekhari et al. [27]. Using this type of methodology with a variant of the ERQ to look at different CR and ES profiles, Zeng et al. [25] found a three-profile solution in a sample of undergraduate nursing students, with (1) a low-ES-use and moderate-CR-use profile (with a prevalence of 6.6%), (2) a moderate-ES-use and high-CR-use profile (89.1%), and (3) a high-ES-use and high-CR-use profile (4.3%). Similarly, Eftekhari et al. [27] conducted a study with a sample of 301 female undergraduate students, and a variant of the ERQ that examined strategy use and capacity. They found four theoretically meaningful emotion regulation profiles, comprising (1) high CR and high ES, (2) high CR and low ES, (3) moderate CR and low ES, and (4) low CR and low ES. Eftekhari et al. [27] compared these profiles in terms of depression, anxiety, and post-traumatic stress disorder symptoms, and indicated that the low-regulator profile had the highest levels of mental health symptoms, whereas the high-CR/low-ES profile had the lowest levels of these symptoms, suggesting that the latter was the most adaptive profile.

Latent profile analysis studies examining a broader range of emotion regulation strategies have also been completed by different research teams (e.g., [28,29,30,31,32,33]), with those studies too highlighting the clinical relevance of different emotion regulation strategy combinations. For instance, van den Heuvel et al. [33] revealed four cognitive emotion regulation strategy profiles in a sample of adolescents. These profiles were (1) Low Regulators (with little use of both adaptive and maladaptive strategies), (2) High Regulators (with the frequent use of both adaptive and maladaptive strategies), (3) Maladaptive Regulators (with little use of adaptive strategies and frequent use of maladaptive ones), and (4) Adaptive Regulators (with average use of adaptive strategies and little use of maladaptive ones) [33]. In that study [33], the Maladaptive profile showed the highest level of depression symptoms compared to other profiles. Such findings indicate the promise of analyzing emotion regulation profiles; therefore, further examinations in larger and diverse samples across different variables are needed.

There is presently a relative lack of studies using a latent profile analysis to investigate emotion regulation strategy profiles (i.e., profiles of CR and ES use) in general community samples, with most work to date having been performed in student samples. There is also a need to examine how such emotion regulation profiles might relate to a broader range of positive and negative mental health outcomes. As such, another key purpose of this paper is to help address these gaps, utilizing both variable-centered and person-centered approaches.

### 1.6. The Present Paper

In this paper, we sought to further explore the latent structure and different latent profiles of emotion regulation, utilizing a Polish general community sample. Specifically, we had two aims.

Our first aim was to examine the latent structure of a Polish version of the ERQ, including an examination of the invariance of this structure across different demographic categories, different levels of psychopathology, and levels of alexithymia. While examining measurement invariance across different demographic categories is a common practice (e.g., testing the structure of the construct across males and females), invariance testing across different levels of psychological constructs is less well known (e.g., testing the structure of a construct across people with different levels of psychopathology) [34]. Emotion regulation and psychopathology are strongly associated [35]; therefore, people with high levels of psychopathology symptoms may understand emotion regulation processes and apply emotion regulation strategies in different ways compared to people without emotional problems (for review, see Dawel et al. [36]). Similarly, as alexithymia is a personality characteristic, comprising deficits in emotion processing [37,38], it may be associated with differential assessment of one’s own emotions and emotion regulation processes. Therefore, it is crucial to reveal whether people assess their emotion regulation strategies similarly regardless of the levels of psychopathology symptoms and alexithymia.

Our second aim was to use the ERQ to investigate profiles of emotion regulation with a latent profile analysis. We examined whether different combinations of CR and ES use exist, and if so, whether these combinations (profiles of emotion regulation) differed in terms of their mental health correlates. We sought to compare the extracted emotion regulation profiles in terms of their relationships with positive (i.e., well-being) and negative (i.e., anxiety and depression symptoms) mental health outcomes, as well as emotion processing difficulties (i.e., alexithymia).

We predicted that the ERQ would have a theoretically congruent factor structure, comprising CR and ES factors [9]. Furthermore, we predicted that profiles characterized by high use of CR and low use of ES would be the most adaptive emotion regulation profiles, demonstrating relationships with more positive outcomes, fewer negative outcomes [1], and lower levels of alexithymia [37].

## 2. Materials and Methods

### 2.1. Procedure

The study was conducted in accordance with the Declaration of Helsinki Ethical Principles. The Ethics Committee of the Faculty of Psychology of Kazimierz Wielki University approved the study (No. 1/13.06.2022, later revision: 28.11.2023). This study and its data have not been published previously.

From November 2023 to May 2024, participants were recruited via announcements placed on Facebook and Instagram. A link with an invitation to complete this online anonymous and voluntary survey (with an appended consent form) was posted. The link was hosted on the Google Forms platform. Before completing the survey, participants provided their written informed consent. Our inclusion criteria were for Polish-speaking people aged 18 years or over, who signed their informed consent for study participation.

### 2.2. Participants

Our sample consisted of 1197 Polish-speaking adults (897 females, 276 males, and 24 non-binary individuals) recruited from the general population in Poland, with ages ranging from 18 to 75 years (*M* = 27.46, *SD* = 11.24). Detailed demographic characteristics of the participants are presented in Table 1. As we examined measurement invariance across two age groups (i.e., aged 18–24 and 25–75), we also presented descriptive statistics specific to these groups.

### 2.3. Measures

#### 2.3.1. The Demographic Questionnaire

All participants filled out a demographic form on their age, sex/gender (females, males, or non-binary individuals), and education level (with four education categories; see Table 1 for details). As this was a self-report study, all the data are based on the information provided by the participants.

All our respondents (*n* = 1197) completed the ERQ and a measure of well-being, and a subset of them (*n* = 529) also completed additional measures of psychopathology symptoms and alexithymia. The full sample was used for the factor analyses and reliability testing, and the subsample was used for all other analyses.

As a sample of around 500 people is regarded as strong for a latent profile analysis [39], our subsample was sufficient for these analyses. The subsample (*n* = 529) consisted of 441 females, 80 males, and 8 non-binary people. The age distribution was *M* = 28.62, *SD* = 12.31, median = 23.00, min. = 18, and max. = 75.

#### 2.3.2. The Emotion Regulation Questionnaire (ERQ) and Its Translation

The ERQ [9] is a 10-item self-report measure of two emotion regulation strategies: CR and ES. Items are scored on a 7-point Likert scale ranging from 1 (“strongly disagree”) to 7 (“strongly agree”). Separate scores are extracted for CR and ES. Higher scores indicate higher usage of these strategies.

In order to develop the Polish version of the ERQ, we asked J.J. Gross [9], one of the developers of the original ERQ, for permission to conduct this translation. We received J.J. Gross’s approval and the recommendations on the ERQ translation.

We followed a standard translation procedure [40]. At the first step of the translation procedure, four independent translators translated the original English version of the ERQ into Polish. Based on these four translations, a common Polish translation was created. During this process, the Polish ERQ items and instructions were discussed. This way, a common Polish translation of the ERQ was developed.

At the second step of the translation procedure, this common Polish version was translated back into English by an independent translator. This back translation was collated with the original English version of the ERQ. The translated version and the original ERQ were judged as consistent. At the third step, this prefinal version of the Polish ERQ was tested within a small group of people spanning various demographic backgrounds (age, gender, education), who were asked to assess the readability of this version of the questionnaire. Minor stylistic and graphical corrections were made, resulting in the final Polish version of the ERQ used in this study. In the Supplementary Materials, we presented this questionnaire with its scoring instructions and Polish norms. This Polish version of the ERQ is freely available for use in research and practice.

#### 2.3.3. The Patient Health Questionnaire-4 (PHQ-4)

The PHQ-4 is a 4-item self-report measure of anxiety and depression symptoms experienced over the previous two weeks [41]. The PHQ-4 has two subscales, each containing two items: the Anxiety subscale (e.g., “Feeling nervous, anxious, or on edge”), and the Depression subscale (e.g., “Feeling down, depressed, or hopeless”). The PHQ-4 Total score, representing an overall level of distress symptoms, can also be computed. All PHQ-4 items are scored on a 4-point Likert scale, ranging from 0 (“not at all”) to 3 (“nearly every day”). Higher scores of the subscales and total scale score indicate higher levels of psychopathology symptoms. A score of ≥3 of the Anxiety and Depression subscales indicates elevated levels of anxiety and depression symptoms, respectively. A score of ≥6 of the PHQ-4 Total score indicates elevated levels of these psychopathology symptoms. The Polish PHQ-4 [42] previously demonstrated good psychometric properties (i.e., strong factorial validity with measurement invariance across age and gender groups, and good internal consistency reliability). For example, the PHQ-4 subscale scores and the total score had reliability of ≥0.73, measured with McDonald’s omega, in a general community sample of Polish females and males. Due to this strong psychometric performance and clinical relevance, the Polish PHQ-4 was used in this study.

#### 2.3.4. The WHO-Five Well-Being Index (WHO-5)

The WHO-5 is a 5-item self-report measure of positive well-being [43,44]. Items (e.g., “I have felt calm and relaxed”) are scored on a 6-point Likert scale, ranging from 0 (“at no time”) to 5 (“all the time”). A higher total score indicates a higher level of well-being. The Polish WHO-5 [45,46] has demonstrated good psychometric properties (i.e., strong factorial validity with measurement invariance across age and gender groups, and good internal consistency reliability). For example, this questionnaire had reliability of 0.85, measured with Cronbach’s alpha and McDonald’s omega, in a general community sample of Poles [45]. Due to these strong psychometric properties and clinical relevance, the Polish WHO-5 was deemed appropriate for this study.

#### 2.3.5. The Perth Alexithymia Questionnaire-Short Form (PAQ-S)

The PAQ-S is a 6-item self-report measure of alexithymia [47]. Items (e.g., “When I’m feeling bad, I can’t tell whether I’m sad, angry, or scared”) are scored on a 7-point scale ranging from 1 (“strongly disagree”) to 7 (“strongly agree”). A higher total scale score indicates a higher level of alexithymia. The Polish PAQ-S [48] has demonstrated good psychometric properties (i.e., strong factorial validity with measurement invariance across age and gender groups, and good internal consistency reliability). For example, this questionnaire had reliability of 0.81, measured with Cronbach’s alpha and McDonald’s omega, in a general community sample of Poles. Due to these strong psychometric properties and clinical relevance, the Polish PAQ-S was used in this study.

### 2.4. Analytic Strategy

Statistical analyses were carried out using *Statistica* v. 13.3 and *R* v. 4.4.0 with the *lavaan* v. 0.6–17 package (for confirmatory factor analysis) as well as the *tidyLPA* v. 1.1.0 package (for latent profile analysis). *JASP* v. 0.18.3 was used for calculating internal consistency reliability and an analysis of covariance (ANCOVA). McDonald’s omega (ω) and Cronbach’s alpha (α) internal consistency reliability coefficients with 95% confidence intervals (CIs) were calculated. Values ≥0.70 were judged as acceptable, ≥0.80 as good, and ≥0.90 as excellent [49].

#### 2.4.1. Factor Structure

A confirmatory factor analysis was used. For this analysis, we applied maximum likelihood estimation with robust standard errors and the Satorra–Bentler scaled test statistic. The theoretically informed two-factor model of the ERQ [9,10] was tested, representing the intended factor structure of the ERQ with CR and ES factors. In this model, items 1, 3, 5, 7, 8, and 10 were specified to load on a CR factor, and items 2, 4, 6, and 9 on an ES factor, with these two factors being allowed to correlate.

The comparative fit index (CFI), Tucker–Lewis index (TLI), root mean square error of approximation (RMSEA), and standardized root mean square residual (SRMR) were used to assess model goodness of fit. CFI and TLI values equal to or larger than 0.90 indicate acceptable fit, and values equal to or larger than 0.95 indicate excellent fit. RMSEA and SRMR values equal to or smaller than 0.08 indicate acceptable fit, and values equal to or smaller than 0.06 indicate excellent fit [50].

#### 2.4.2. Measurement Invariance

The measurement invariance testing of the ERQ’s factor structure was performed across several demographic categories: for gender (females vs. males), age (younger people aged 18–24 vs. older people aged 25–75), and education level (no university degree vs. university degree; see Table 1). Regarding age, we divided our sample into the two above-described age groups based on common groupings in the United Nations classification [51].

We also examined measurement invariance across different levels of psychopathology symptoms (PHQ-4 Total scores of 0–5 vs. PHQ-4 Total scores of 6–12, as a PHQ-9 total score of ≥6 indicates clinically elevated levels of psychopathology symptoms [41] and alexithymia (elevated alexithymia vs. non-elevated alexithymia; a 75th percentile of PAQ-S scores [i.e., a raw score of 27 or more] in the study sample was used as the criterion for splitting the sample).

Configural, metric, and scalar invariance models were tested, to determine the extent to which the structure of the ERQ was similar or different across the groups. The following measurement invariance criteria were used: an absolute difference in CFI equal to or smaller than 0.01 for indicating invariance in all invariance models (i.e., metric and scalar), and absolute differences in RMSEA equal to or smaller than 0.015 and in SRMR equal to or smaller than 0.030 for indicating metric invariance, as well as with absolute differences in RMSEA equal to or smaller than 0.015 and in SRMR equal to or smaller than 0.010 for indicating scalar invariance (see [52,53]).

#### 2.4.3. Relationships with Positive and Negative Mental Health Outcomes

For assessing the concurrent validity of ERQ scores and relationships with external markers, Pearson correlations between ERQ scores, PHQ-4 scores (anxiety and depression symptoms), WHO-5 scores (well-being), and PAQ-S scores (alexithymia) were computed.

#### 2.4.4. Demographic Differences

Pearson correlations between ERQ scores and age in groups of females and males were separately calculated. We compared the ERQ scores between (1) females and males, and (2) between people without a university degree and people with a university degree, using an ANCOVA with age as a covariate (to control the potential effects of age). In this study, for gender, the sample of people identifying as non-binary was too small to meet statistical assumptions (*n* = 24), and therefore this sample was not included in the gender comparisons. However, descriptive statistics for the non-binary group were included.

#### 2.4.5. Latent Profile Analysis

Using the *tidyLPA* v. 1.1.0 package in *R* v. 4.4.0 software, we conducted a latent profile analysis [54]. To explore different potential profiles of emotion regulation, our latent profile analysis included the two ERQ scores (CR and ES) as the variables of interest. We used the default model parameters (Model 1: equal variances, covariances fixed to 0), and evaluated and compared solutions from 1 to 8 profiles. If a solution with 8 profiles proved to be superior, the analysis would be repeated with a higher number of profiles to see if a higher count of profiles would yield a better fit.

We based our latent profile analysis evaluation on five common fit index values: the Akaike Information Criterion (AIC), Bayesian Information Criterion (BIC), Classification Likelihood Criterion (CLC), Kullback Information Criterion (KIC), and Appropriate Weight of Evidence Criterion (AWE). Lower values for all these fit indices indicate a better fitting model [39,55,56]. Also, we presented entropy values, which range from 0 to 1, with higher values indicating a higher confidence for classifying participants into the extracted profiles. Entropy values of ≥0.60 are considered acceptable [39].

We also applied an analytic hierarchy process [55], which compares across multiple fit index values, to determine the optimal number of profiles explaining the data. Collectively, this approach aligns with common recommendations for the conducting of a latent profile analysis (see Spurk et al. [39]).

#### 2.4.6. Profile Comparisons

After extracting the profiles of CR and ES from the latent profile analysis, to help determine the relative adaptiveness or maladaptiveness of the profiles, we compared the extracted emotion regulation profiles in terms of whether they differed across positive (well-being) and negative affective outcomes (anxiety and depression symptoms), and alexithymia levels.

#### 2.4.7. Group Norms

We calculated Bayesian percentile rank norms [57,58] for the ERQ subscale scores, with 90% and 95% confidence intervals for the total sample, and females and males separately.

## 3. Results

### 3.1. Descriptive Statistics and Internal Consistency Reliability Coefficients

Descriptive statistics and internal consistency reliability coefficients for all the study variables are presented in Table 2.

The two ERQ scale scores had good internal consistency reliability, with ω and α equal to or larger than 0.77. All other administered measures also showed good reliability, with ω and α coefficients of 0.78 and larger.

### 3.2. Factor Structure

Descriptive statistics for all ERQ items are displayed in Table 3.

The intended two-factor correlated ERQ model was a good fit according to CFI, but was initially below some of the cut-offs for a good fit on other indices. Modification indices indicated that a correlated error term should be added between items 1 and 3. Based on past work on the ERQ’s psychometrics (e.g., [10,59,60]), and content similarities within items 1 and 3, we felt that adding this correlated error term to the model was theoretically justified. With the correlated error term added, model fit for the two-factor model was strong, with fit indices in the good or excellent range (see Table 3). In this final two-factor model with a correlated error term between items 1 and 3, standardized factor loadings of all ERQ items were salient on their intended factor (from 0.46 to 0.86, all *ps* < 0.001; see Table 3), thus indicating good performance for all items. The estimated correlation between CR and ES was 0.00 (*p* > 0.05), indicating that the two ERQ factors were uncorrelated.

### 3.3. Measurement Invariance

We tested the configural, metric, and scalar invariance of the final model (i.e., the two-factor model with a correlated error term between items 1 and 3) across gender, age, and education categories, as well as across psychopathology symptom levels and alexithymia levels (Table 4).

In all these invariance analyses for the demographic categories, differences in CFI values were less than an absolute value of 0.01. These results therefore supported full metric and scalar invariance for the ERQ across the gender, age, and education categories. In terms of the other fit indices used in our measurement invariance analysis, the differences in RMSEA and SRMR values further strongly supported full metric and scalar invariance across all the examined demographic groups.

In terms of invariance across different levels of psychopathology symptoms and alexithymia, the differences in CFI values were less than an absolute value of 0.01, indicating that metric invariance was supported. The differences in CFI values were slightly higher than an absolute value of 0.01 for scalar invariance testing (i.e., −0.012 and −0.013 for psychopathology symptoms and alexithymia levels, respectively), whereas all other fit indices (i.e., RMSEA and SRMR) were strong in their support of invariance. Taking into account all the fit indices, and only slight excesses of the threshold value for CFI, on balance, we think it can be concluded that scalar invariance was supported for ERQ scores across psychopathology symptoms and alexithymia levels.

### 3.4. Relationships with Positive and Negative Mental Health Outcomes

In our Pearson correlational analyses (see Table 5), we found that the ERQ CR score was negatively correlated with anxiety and depression symptoms (*r* from −0.23 to −0.28, all *ps* < 0.001), and alexithymia (*r* = −0.21, *p* < 0.001), as well as positively correlated with well-being (*r* = 0.39, *p* < 0.001).

In contrast, the ERQ ES score was positively correlated with anxiety and depression symptoms (*r* from 0.18 to 0.34, all *ps* < 0.001), and alexithymia (*r* = 0.51, *p* < 0.001), as well as negatively correlated with well-being (*r* = −0.26, *p* < 0.001). These patterns support predictions of CR being a generally adaptive strategy, and ES being generally maladaptive.

### 3.5. Demographic Differences

We calculated Pearson correlations between the two ERQ scale scores and age, for females and males separately. In females, age was positively correlated with CR (*r* = 0.13, *p* < 0.001), whereas age was negatively correlated with ES (*r* = −0.12, *p* < 0.001). In males, there were no statistically significant correlations between age and the two ERQ strategies (*r* = 0.10 and *p* = 0.089 for CR; *r* = −0.08 and *p* = 0.192 for ES).

We used two sets of ANCOVAs (with age used as a covariate) to compare ERQ scores across two gender categories (females vs. males) and two education levels (no university degree vs. university degree). We found statistically significant gender differences in CR scores, *F*_(1, 1170)_ = 6.35, *p* = 0.012, η^2^ = 0.005; males reported using CR more often than females. There were also statistically significant gender differences in ES scores, *F*_(1, 1170)_ = 28.86, *p* < 0.001, η^2^ = 0.024; males reported using ES more often than females. CR and ES use did not differ significantly across the education categories (*p* > 0.05).

### 3.6. Latent Profile Analysis

**Figure 1:**
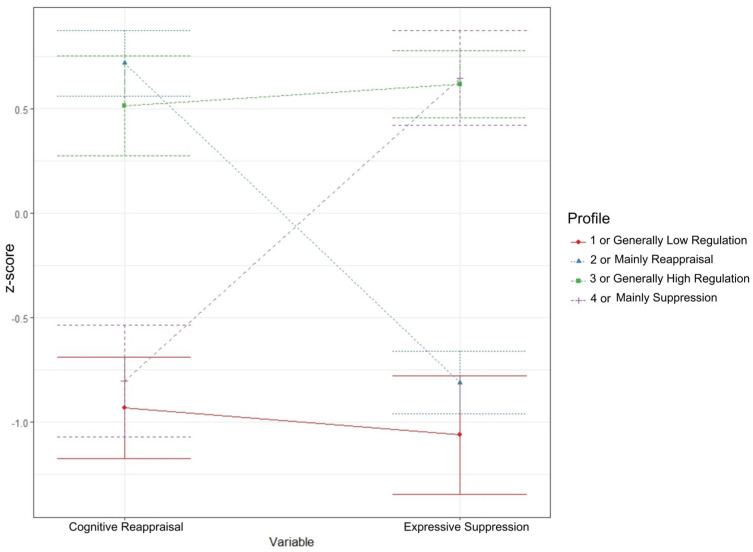
Latent profile analysis of cognitive reappraisal and expressive suppression use on ERQ (*n* = 529).

In our latent profile analysis, the data were best represented by a four-profile solution (see Figure 1).

Fit index values for all solutions are provided in Appendix A. According to the analytic hierarchy process [55], this four-profile solution was the most optimal. This four-profile solution highlighted meaningfully different profiles in terms of patterns of CR and ES use. Profile 1 (which we call the *Generally Low Regulation* profile) comprised people with low CR and low ES scores. Profile 2 (which we call the *Mainly Reappraisal* profile) was composed of people with high CR and low ES scores. Profile 3 (which we call the *Generally High Regulation* profile) comprised people with high CR and high ES scores. Lastly, Profile 4 (which we call the *Mainly Suppression* profile) comprised people with low CR and high ES scores.

### 3.7. Profile Comparisons

Using ANOVA with post hoc comparisons (see Table 6 for means in each profile group), we compared the four extracted profiles in terms of their anxiety and depression symptoms, well-being, and alexithymia levels.

We found that the most favorable mental health outcomes (i.e., low psychopathology levels and high well-being levels) and the lowest alexithymia levels were present in the Mainly Reappraisal profile (Profile 2), whereas the most unfavorable mental health outcomes (i.e., high psychopathology levels and low well-being levels) and the highest alexithymia levels were present in the Mainly Suppression profile (Profile 4). In the Mainly Suppression profile, the mean anxiety and depression scores were above the cut-off score of 3, indicating elevated symptoms, and levels of alexithymia were also highest in this profile.

The Generally Low Regulation (Profile 1) and Generally High Regulation (Profile 3) profiles were generally similar in their levels of mental health symptoms, with no significant differences between these profiles in depression, anxiety, or well-being levels. Psychopathology symptoms were elevated (anxiety scores were above the cut-off of 3 in both profiles, and depression scores were above the cut-off of 3 in the Generally High Regulation profile), though not as highly as in the Mainly Suppression profile (Profile 4). Levels of alexithymia were higher in the Generally High Regulation profile (compared to the Generally Low Regulation profile), with similar levels to those seen in the Mainly Suppression profile.

### 3.8. Group Norms

Polish norms for the ERQ were computed and are presented in the Supplementary Materials.

## 4. Discussion

Our aim in this study was to introduce and validate a Polish ERQ, and use it to examine the latent structure and profiles of emotion regulation in terms of CR and ES usage. Overall, our results show that the ERQ is a strong measure of CR and ES strategies, that people can utilize different combinations of these strategies, and that such patterns have important implications for mental health.

### 4.1. Latent Structure

In our data set, we found the latent structure of the ERQ to be well represented by its intended two-factor structure, corresponding to CR and ES factors. These findings are in line with the large body of previous findings on the psychometrics on the ERQ in various language versions [10,12,13,14,15,16,17,18], and findings for its six-item short version, the ERQ-S [11,19]. Similarly to previous research (e.g., [9,15,17]), we noted that the CR and ES factors were uncorrelated, indicating that these two strategies are largely orthogonal to each other. This orthogonality provides high potential for different combinations of the strategies to exist across people (i.e., people with high or low levels of the respective strategies), as we will later discuss with respect to our latent profile analysis results.

### 4.2. Internal Consistency Reliability

These two factors of the Polish ERQ also had good internal consistency reliability, thus supporting the robustness of the extracted scores. This is also consistent with past work on the ERQ (e.g., [9,10,13,15]), and alongside the factor analytic results, further supports the cross-cultural applicability of the questionnaire (Burghart et al. [61], Chan et al. [62], Van Doren et al. [63]).

### 4.3. Measurement Invariance

Importantly, our results demonstrate that the ERQ’s two-factor structure was highly consistent across different demographic groups and groups with different psychological characteristics. Our analyses empirically supported scalar invariance of the ERQ across females and males, and younger and older people, as well as across people with a university degree and those without a university degree, which aligns with past reports (e.g., García et al. [8], Brady et al. [13], Pinto et al. [16], Preece et al. [17], Zhang and Bian [64]). As previous studies indicated good psychometric properties of the ERQ in clinical samples (e.g., Andrea et al. [59], Brandão et al. [60]), we were also interested in further examining whether the ERQ performs well and similarly between people with heightened psychological distress (i.e., individuals with elevated psychopathology symptoms) or those with emotion processing difficulties (i.e., high alexithymia; Preece et al. [37]) and those without difficulties in these areas. In this regard, we also noted scalar invariance, indicating that the assessment of emotion regulation strategies via the ERQ seems to be valid regardless of people’s levels of psychopathology symptoms and alexithymia. This helps to support the broad utility of the ERQ and its validity in a range of group comparisons.

### 4.4. Demographic Differences

Given our demonstration of invariance and strong psychometric performance, we sought to use the Polish ERQ to understand more about emotion regulation differences between different demographic groups. In light of previous studies indicating specific relationships between age and emotional variables across genders (e.g., Masumoto et al. [65]), we examined whether the two ERQ emotion regulation strategies were associated with age in females and males separately. In females, age was slightly positively correlated with CR and negatively correlated with ES, whereas in males, there were no statistically significant correlations between age and the two ERQ strategies. These results are in accordance with previous Polish studies, indicating a shift to more favorable emotional functioning with age in females, but not in males (e.g., [42,45]). However, this finding is not always replicated across cultures (e.g., Masumoto et al. [65]).

As for gender differences, we noted that males reported using CR and ES more often than females; however, the effect size of these differences was small, especially for CR scores. A significantly higher level of ES in males was also noted in past research (e.g., Pinto et al. [16], Olalde-Mathieu et al. [66], Zhang and Bian [64]), whereas conclusions on gender differences in CR scores tend to be mixed (e.g., Masumoto et al. [65], Nolen-Hoeksema [67], Seixas et al. [68]). Also, in line with previous reports (Nakagawa et al. [69]), CR and ES scores did not differ significantly across education categories in our study.

### 4.5. Relationships with Positive and Negative Mental Health Outcomes

Overall, our results empirically supported the clinical relevance of assessing CR and ES, as both strategies were related to mental health outcomes. Patterns of correlations supported previous findings that CR can generally be considered an adaptive strategy, and ES a maladaptive strategy [9,10,12,14,17,18,19,70]. In our data, use of CR was robustly associated with better well-being, lower depression and anxiety, and lower alexithymia. The opposite pattern was present for ES, being robustly associated with poorer well-being, high depression and anxiety, and higher alexithymia.

### 4.6. Latent Profiles of Emotion Regulation

Novelly, our latent profile analysis highlighted the heterogeneity that can be present across people, with different subgroups using different combinations of these strategies. We extracted four theoretically meaningful profiles. Some people appear to rely mainly on CR to regulate their emotions (Mainly Reappraisal profile; 28.17% of our sample), some mainly on ES (Mainly Suppression profile; 29.68% of our sample), some on neither of these strategies (Generally Low Regulation profile; 11.91% of our sample), and some on both of these strategies (Generally High Regulation profile; 30.25% of our sample). Importantly, we found that these emotion regulation profiles were linked to different mental health outcomes. Consistent with the adaptive status of CR, and the maladaptive status of ES, the Mainly Reappraisal profile clearly had the best mental health outcomes, and the Mainly Suppression profile had the worst. Such findings highlight the utility of including a prominent role for CR within one’s habitual ways of managing emotions [9,71], and highlight the dangers of regulation patterns that focus principally on avoidance and suppression [9,70,72]. Taken together, our findings therefore support the specifications of the *process model of emotion regulation* [1,6], that different emotion regulation strategies should have different effects on down-stream affective outcomes, and that CR is likely to be more adaptive than ES [9].

There is also an interesting contrast with those profiles that had high use of both strategies (Generally High Regulation profile) and those with low use of both strategies (Generally Low Regulation profile). These profiles had similar levels of mental health issues, with scores that indicated some elevated mental ill-health, though not reaching the levels of severity of the Mainly Suppression profile. With respect to the Generally High Regulation profile, it may be that the use of ES predisposes them to poor outcomes over time [9], but the integration of CR helps to temper this somewhat (relative to profiles that use only ES). It is also possible that people in the Generally High Regulation profile use many strategies, but their application of the strategies is not necessarily targeted and context-appropriate (e.g., a “scatter gun approach”), and is thus suboptimal [73]. This Generally High Regulation profile also had heightened levels of alexithymia, suggesting a generally disengaged and avoidant approach to emotions. Alexithymia inhibits understanding of emotions, and thus the capacity to match strategy approaches to specific emotions and contexts (see *attention-appraisal model of alexithymia*; Preece et al. [37]). In such instances, high use of a range of strategies (even if some are technically adaptive strategies) may not be optimally helpful (see also Pinto et al. [24]). Regarding the Generally Low Regulation profile, given their elevated mental health symptoms, it may be that their lack of attempts to down-regulate a negative affect (at least using CR or ES) contributes to those elevated symptoms. However, we did only measure CR and ES, and people do use strategies outside of those two [1], so it is also possible that the Generally Low Regulation profile (and other profiles) may rely on other non-measured strategies.

### 4.7. Future Directions

These profiles we found have some broad correspondence with the small number of previous studies that have examined latent profiles of emotion regulation strategy use. Such studies commonly reveal profiles characterized by reliance on generally adaptive strategies, reliance on avoidant and maladaptive strategies, a mix of both, or very little use of any strategies (e.g., Pinto et al. [24], Zeng et al. [25], Eftekhari et al. [27]). Further work of this type will be important to continue exploring person-level individual differences in emotion regulation, and how different combinations of strategies exist and may facilitate or impair long-term mental health. It will be important to perform such work in samples with diverse demographic profiles, for example, allowing the examination of how relationships may differ between females, males, and those with a non-binary gender.

We think a focus on CR and ES, as well as studied and contrasted strategy categories, was useful here, but in future work, it will also be useful to build on these findings by including a wider range of emotion regulation strategies. For example, one might include strategies across all five stages of the process model of emotion regulation (Olderbak et al. [74]).

### 4.8. Practical Implications of the Study and the Use of Polish Norms

Our findings have several important implications for the assessment and treatment of issues in emotion regulation. Regarding treatment, our findings support that mental health treatment approaches focused on increasing CR and decreasing ES are likely to have strong utility for improving affective outcomes (e.g., Gratz et al. [2], Wang and Yin [71]). This is a focus often featuring in cognitive behavioral therapy (CBT) protocols, where patients are taught to reappraise unhelpful thinking styles, and develop skills to express their emotions to others in healthier ways [75,76]. As such, our findings support the use of these types of CBT approaches to facilitate better affective outcomes. Our findings also indicate that those with maladaptive emotion regulation patterns will also often have difficulty understanding their emotions (i.e., high alexithymia), thus highlighting the potential value of addressing both emotion regulation and alexithymia in treatment (see Preece et al. [37]).

In terms of assessment, our findings suggest that there is likely value in routinely assessing emotion regulation (via validated tools like the ERQ), in order to identify people where their emotion regulation profile may place them at risk for poor affective outcomes. As we have presented norms for the Polish ERQ for the total sample, as well as females and males separately, this should help to support the use and interpretation of the ERQ in various settings, including clinical settings.

In our Polish norms, corresponding to standard interpretative conventions, percentile rank scores of ≤15 are indicative of a *low* level of use of CR or ES, whereas percentile rank scores from 16 to 84 are indicative of an *average* level of use, and scores of ≥85 are indicative of a *high* level of strategy use. As aforementioned, low levels of CR and/or high levels of ES are likely to indicate maladaptive patterns of emotion regulation [9], and therefore people with such profiles are at a higher risk of poor affective outcomes.

### 4.9. Limitations

Some limitations of our study should be noted that will require further research. Firstly, this was a cross-sectional study and therefore conclusions regarding the cause-and-effect relationships between emotion regulation strategy use and other study variables cannot be established. In cases where we have speculated on directionality, this has been informed by the consistency of results with theoretical predictions (e.g., with the process model of emotion regulation [1]).

Our study, by design, was performed with a Polish sample; therefore, the results are most relevant to the Polish population. For instance, the prevalence of mental health issues is quite high in Poland (see [42,45]), meaning that our results may not generalize to other cultures. More cross-cultural work in the future will be an important direction, and we feel that the Polish ERQ introduced here can be a useful tool for such work to include Polish-speaking populations. Indeed, other work demonstrating the cross-cultural invariance of the ERQ (e.g., Van Doren et al. [63], Sala et al. [77]) highlights the high potential of the ERQ for cross-cultural research.

Our study recruitment was for a voluntary and anonymous online survey, and thus was vulnerable to self-selection bias. However, we tried to mitigate this limitation by diverse sampling across multiple social networking sites. Our sample was not fully representative of the Polish population (e.g., the gender ratio was not equal), but nonetheless was relatively large in size overall (*n* = 1197) and consisted of a range of demographic backgrounds. It was therefore sufficient for calculating norms and the complex statistical analyses we conducted (e.g., latent profile analysis). Future studies with large sample sizes could look in more detail at emotion regulation patterns across various demographic groups (e.g., across gender categories). Our sample was also not a clinical sample. Therefore, future work in clinical settings will also be beneficial to test the generalizability of our findings.

Lastly, all the measures we administered to examine relationships with external outcome variables (e.g., well-being, mental health) were self-report measures. These measures are all well validated and commonly used in the field, but future work with behavioral, observer-rated, or lab-based markers of these constructs will also be useful.

## 5. Conclusions

Our data show that the Polish ERQ is a robust measure of emotion regulation, and that its latent structure is well represented by separate CR and ES factors. These emotion regulation strategies appear to have high clinical relevance, supporting the predictions of the process model of emotion regulation [1]. Our findings highlight that people can present with different combinations (i.e., profiles) of strategy usage, and that such profiles show important linkages with well-being and mental health outcomes. The ERQ therefore seems to be a useful tool to inform research and clinical practice.

## Figures and Tables

**Table 1:** Demographic characteristics of the participants.

Demographic Characteristics			*n*	%
Age		*M* = 27.46, *SD* = 11.24, median = 23.00, min. = 18, max. = 75	1197	100
	People aged 18–24	*M* = 20.60, *SD* = 1.86, median = 20.00, min. = 18, max. = 24	720	60.15
	People aged 25–75	*M* = 37.81, *SD* = 11.56, median = 35.00, min. = 25, max. = 75	477	39.85
Gender		Females	897	74.94
		Males	276	23.06
		Non-binary individuals	24	2.01
Education	University degree	Higher	410	34.25
	No university degree (total *n* = 787)	Secondary	661	55.22
		Vocational	63	5.26
		Primary	63	5.26

**Table 2:** Descriptive statistics and internal consistency reliability coefficients for the study variables.

Scale/Subscale	Total Sample					Females			Males			Non-Binary		
	*n*	ω (95% CI)	α (95% CI)	*M*	*SD*	*n*	*M*	*SD*	*n*	*M*	*SD*	*n*	*M*	*SD*
ERQ Cognitive reappraisal	1197	0.86 (0.85; 0.87)	0.86 (0.84; 0.87)	25.01	8.16	897	24.64	8.15	276	26.29	8.09	24	24.25	8.24
ERQ Expressive suppression	1197	0.79 (0.77; 0.81)	0.77 (0.75; 0.79)	16.33	5.88	897	15.84	6.01	276	17.84	5.20	24	17.25	5.70
PHQ-4 Anxiety	529	0.78 (0.74; 0.82)	0.78 (0.74; 0.82)	3.50	1.82	441	3.59	1.78	80	2.93	2.00	8	4.50	1.41
PHQ-4 Depression	529	0.83 (0.80; 0.86)	0.83 (0.80; 0.86)	2.95	1.95	441	3.02	1.96	80	2.59	1.89	8	3.25	1.83
PHQ-4 Total score	529	0.86 (0.84; 0.88)	0.86 (0.84; 0.88)	6.45	3.46	441	6.60	3.40	80	5.51	3.72	8	7.75	3.01
WHO-5 Total score	1197	0.85 (0.84; 0.86)	0.85 (0.84; 0.86)	8.99	4.78	897	8.59	4.61	276	10.50	5.08	24	6.71	3.74
PAQ-S Total score	529	0.83 (0.81; 0.85)	0.83 (0.81; 0.85)	20.71	8.79	441	20.75	8.84	80	19.99	8.54	8	25.63	7.60

**Table 3:** Descriptive statistics of the ERQ items and standardized factor loadings from the confirmatory factor analysis of the 2-factor correlated model with an error term between items 1 and 3 (*n* = 1197).

ERQ Items	*M*	*SD*	Skewness	Kurtosis	Factor Loading on Intended Factor	
1. “When I want to feel more positive emotion (such as joy or amusement), I *change what I’m thinking about*.”	4.34	1.75	−0.37	−0.76	0.56	–
2. “I keep my emotions to myself.”	4.86	1.88	−0.58	−0.85	–	0.75
3. “When I want to feel less *negative* emotion (such as sadness or anger), I *change what I’m thinking about*.”	4.13	1.86	−0.14	−1.06	0.63	–
4. “When I am feeling *positive* emotions, I am careful not to express them.”	2.67	1.72	0.94	−0.11	–	0.46
5. “When I’m faced with a stressful situation, I make myself *think about it* in a way that helps me stay calm.”	4.42	1.91	−0.35	−1.02	0.59	–
6. “I control my emotions by *not expressing them*.”	4.23	2.05	−0.24	−1.27	–	0.81
7. “When I want to feel more *positive* emotion, I *change the way I’m thinking* about the situation.”	4.17	1.72	−0.23	−0.83	0.79	–
8. “I control my emotions by *changing the way I think* about the situation I’m in.”	3.97	1.73	−0.17	−0.92	0.78	–
9. “When I am feeling *negative* emotions, I make sure not to express them.”	4.57	1.96	−0.43	−1.08	–	0.70
10. “When I want to feel less *negative* emotion, I *change the way I’m thinking* about the situation.”	3.99	1.72	−0.16	−0.93	0.86	–

*Note*. All factor loadings are statistically significant (*ps* < 0.001).

**Table 4:** Goodness-of-fit index values for the 2-factor models in the total sample, and measurement invariance.

Sample/Model	χ^2^ (df)	CFI	TLI	RMSEA (90% CI)	SRMR	ΔCFI	ΔRMSEA	ΔSRMR	Invariance Testing
Total sample (*n =* 1197)/2-factor model	374.92 (34)	0.908	0.879	0.103 (0.094; 0.112)	0.069	–	–	–	–
Total sample (*n =* 1197)/2-factor model with a correlated error term between items 1 and 3	254.94 (33)	0.941	0.92	0.084 (0.074; 0.093)	0.063	–	–	–	–
Invariance models (for the final 2-factor model with an error term between items 1 and 3)									
*Gender invariance (females [n = 897]* vs. *males [n = 276])*									
Configural	292.56 (66)	0.939	0.916	0.085 (0.075; 0.095)	0.059	–	–	–	–
Metric	303.75 (74)	0.939	0.926	0.080 (0.071; 0.090)	0.06	0	−0.005	0.001	Supported
Scalar	319.16 (82)	0.938	0.932	0.077 (0.068; 0.085)	0.061	−0.001	−0.003	0.001	Supported
*Age invariance (aged 18–24 [n = 720]* vs. *aged 25–75 [n = 477])*									
Configural	295.04 (66)	0.939	0.917	0.085 (0.075; 0.095)	0.06	–	–	–	–
Metric	310.24 (74)	0.939	0.925	0.081 (0.072; 0.090)	0.062	0	−0.004	0.002	Supported
Scalar	332.55 (82)	0.936	0.93	0.078 (0.069; 0.087)	0.063	−0.003	−0.003	0.001	Supported
*Education invariance (no university degree [n = 787]* vs. *university degree [n = 410])*									
Configural	297.86 (66)	0.939	0.917	0.085 (0.075; 0.095)	0.061	–	–	–	–
Metric	313.42 (74)	0.939	0.925	0.081 (0.072; 0.090)	0.063	0	−0.004	0.002	Supported
Scalar	334.27 (82)	0.937	0.93	0.078 (0.069; 0.087)	0.064	−0.002	−0.003	0.001	Supported
*Psychopathology symptom invariance (PHQ-4 Total scores of 0–5 [n = 234]* vs. *PHQ-4 Total scores of 6–12 [n = 295])*									
Configural	156.44 (66)	0.947	0.928	0.079 (0.063; 0.095)	0.059	–	–	–	–
Metric	163.68 (74)	0.948	0.937	0.073 (0.058; 0.089)	0.062	0.001	−0.006	0.003	Supported
Scalar	194.59 (82)	0.936	0.93	0.077 (0.063; 0.091)	0.068	−0.012	0.004	0.006	Supported
*Alexithymia level invariance (PAQ-S Total scores of 6–26 [n = 391]* vs. *PAQ-S Total scores of 27–42 [n = 138])*									
Configural	156.58 (66)	0.944	0.923	0.080 (0.064; 0.096)	0.06	–	–	–	–
Metric	167.60 (74)	0.942	0.929	0.077 (0.061; 0.092)	0.066	−0.002	−0.003	0.006	Supported
Scalar	199.76 (82)	0.929	0.922	0.081 (0.066; 0.095)	0.072	−0.013	0.004	0.006	Supported

*Note*. χ^2^ = chi-square statistic; df = degrees of freedom; 90% CI = 90% confidence interval.

**Table 5:** Pearson correlations between the ERQ scores and the other study variables.

Variables	1	2	3	4	5	6	7
1. ERQ Cognitive reappraisal (*n* = 1197)	—						
2. ERQ Expressive suppression (*n* = 1197)	−0.03	—					
3. PHQ-4 Anxiety (*n* = 529)	−0.23 ***	0.18 ***	—				
4. PHQ-4 Depression (*n* = 529)	−0.28 ***	0.34 ***	0.69 ***	—			
5. PHQ-4 Total score (*n* = 529)	−0.28 ***	0.28 ***	0.91 ***	0.92 ***	—		
6. WHO-5 Total score (*n* = 1197)	0.39 ***	−0.26 ***	−0.65 ***	−0.73 ***	−0.75 ***	—	
7. PAQ-S Total score (*n* = 529)	−0.21 ***	0.51 ***	0.23 ***	0.36 ***	0.32 ***	−0.35 ***	—

*Note*. *** *p* < 0.001.

**Table 6:** Comparative analysis results of the examined variables across the emotion regulation profiles.

	Generally Low Regulation or Profile 1 (*n* = 63)	Mainly Reappraisal or Profile 2 (*n* = 149)	Generally High Regulation or Profile 3 (*n* = 160)	Mainly Suppression or Profile 4 (*n* = 157)	ANOVA Parameters	Significant Differences Between Profiles (Post Hoc Comparisons)	Effect Size (η^2^)
Profile Prevalence (%)	11.91	28.17	30.25	29.68			
Profile Description	Low CR with Low ES	High CR with Low ES	High CR with High ES	Low CR with High ES			
Variables	*M* (*SD*)	*M* (*SD*)	*M* (*SD*)	*M* (*SD*)			
1. ERQ Cognitive reappraisal	15.92 (4.24)	31.09 (5.00)	30.19 (4.62)	17.37 (4.69)	*F*_(3, 525)_ = 363.29, *p* < 0.001	1 < 2, 1 < 3, 2 > 4, 3 > 4	0.67
2. ERQ Expressive suppression	8.75 (2.95)	10.66 (2.97)	20.18 (3.16)	19.58 (3.72)	*F*_(3, 525)_ = 386.38, *p* < 0.001	1 < 2, 1 < 3, 1 < 4, 2 < 3, 2 < 4	0.69
3. PHQ-4 Anxiety	3.62 (1.81)	2.95 (1.81)	3.51 (1.67)	3.96 (1.86)	*F*_(3, 525)_ = 8.36, *p* < 0.001	2 < 3, 2 < 4	0.05
4. PHQ-4 Depression	2.84 (1.82)	2.05 (1.80)	3.13 (1.82)	3.68 (1.92)	*F*_(3, 525)_ = 20.66, *p* < 0.001	1 > 2, 1 < 4, 2 < 3, 2 < 4, 3 < 4	0.11
5. PHQ-4 Total score	6.46 (3.42)	4.99 (3.33)	6.64 (3.23)	7.64 (3.37)	*F*_(3, 525)_ = 16.49, *p* < 0.001	1 > 2, 2 < 3, 2 < 4, 3 < 4	0.09
6. WHO-5 Total score	8.79 (4.81)	11.36 (4.81)	8.90 (4.50)	7.11 (4.23)	*F*_(3, 525)_ = 22.42, *p* < 0.001	1 < 2, 2 > 3, 2 > 4, 3 > 4	0.11
7. PAQ-S Total score	17.65 (8.45)	15.05 (6.73)	23.82 (7.66)	24.14 (8.61)	*F*_(3, 525)_ = 47.94, *p* < 0.001	1 < 3, 1 < 4, 2 < 3, 2 < 4	0.22

*Note*. Post hoc comparisons were computed using Tukey’s test, with a *p*-level of 0.05 treated as a statistically significant level.

## Data Availability

The raw data supporting the conclusions of this article will be made available by the authors on request.

## Supplementary Materials

The following supporting information can be downloaded at: https://www.mdpi.com/article/10.3390/jcm14020587/s1, Table S1: Percentile ranks norms for CR scores in the total sample (*n* = 1197). Table S2: Percentile ranks norms for ES scores in the total sample (*n* = 1197). Table S3: Percentile ranks norms for CR scores in the female sample (*n* = 897). Table S4: Percentile ranks norms for ES scores in the female sample (*n* = 897). Table S5. Percentile ranks norms for CR scores in the male sample (*n* = 276). Table S6. Percentile ranks norms for ES scores in the male sample (*n* = 276).

## Appendix A

**Table A1:** Fit index values for latent profile analysis (*n* = 529).

Model	Profiles	AIC	BIC	CLC	KIC	AWE	Entropy
1	1	3008.47	3025.56	3002.47	3015.47	3060.64	1
1	2	2995.96	3025.86	2982.98	3005.96	3089.74	0.51
1	3	2990.55	3033.26	2971.38	3003.55	3125.14	0.41
1	4	2975.29	3030.81	2950.43	2991.29	3150.2	0.57
1	5	2972.55	3040.88	2941.77	2991.55	3188	0.61
1	6	2973.82	3054.97	2937.01	2995.82	3229.93	0.6
1	7	2980.47	3074.43	2937.58	3005.47	3277.29	0.56
1	8	2985.93	3092.7	2937.02	3013.93	3323.39	0.54

*Note*. An analytic hierarchy process, based on the fit indices AIC, AWE, BIC, CLC, and KIC [55], suggests that the best solution is Model 1 with 4 profiles.
