# Supplementary material for: Latent Structure and Profiles of Emotion Regulation: Reappraisal and Suppression Patterns with the Polish Version of the Emotion Regulation Questionnaire
Source: J Clin Med. 2025 Jan 17;14(2):587. doi: 10.3390/jcm14020587 (PMC11765774; doi:10.3390/jcm14020587)
Supplement: Supplementary file 1 [file jcm-14-00587-s001.zip › jcm-3330594-supplementary.pdf]

Supplementary Table S1. Percentile ranks norms for CR scores in the total sample (n = 1197).

Raw Score	Percentile Rank	95% Interval		90% Interval	
		Lower	Upper	Lower	Upper
6	0.8	0.0	1.9	0.0	1.7
7	1.9	1.1	2.9	1.2	2.7
8	2.8	1.8	4.1	1.9	3.9
9	3.9	2.8	5.2	2.9	5.0
10	4.9	3.6	6.5	3.8	6.2
11	6	4.6	7.4	4.8	7.2
12	7	5.5	9.3	5.8	8.9
13	9	7.2	10.7	7.5	10.4
14	10	8.4	12.2	8.6	11.9
15	12	10.0	14.9	10.4	14.5
16	15	12.6	17.4	13.0	17.0
17	17	14.8	20.1	15.2	19.7
18	20	17.5	23.5	18.0	23.0
19	24	20.8	27.0	21.3	26.5
20	27	24.2	30.5	24.6	30.0
21	30	27.4	33.7	27.9	33.2
22	34	30.6	37.7	31.1	37.1
23	38	34.7	41.8	35.2	41.2
24	43	38.8	46.6	39.4	46.1
25	48	43.7	51.4	44.3	50.9
26	52	48.5	56.0	49.0	55.5
27	57	53.1	60.7	53.6	60.1
28	62	57.8	65.3	58.3	64.8
29	66	62.4	69.6	63.0	69.0
30	70	66.8	74.0	67.3	73.5
31	75	71.5	78.4	72.0	77.9
32	79	75.9	82.0	76.3	81.6
33	83	79.7	85.6	80.1	85.2
34	86	83.5	89.0	84.0	88.6
35	89	87.0	91.5	87.4	91.2
36	92	89.6	93.8	89.9	93.5
37	94	92.2	95.6	92.4	95.3
38	95.4	94.0	96.6	94.2	96.4
39	96.7	95.2	97.9	95.4	97.8
40	97.8	96.7	98.6	96.9	98.5
41	98.3	97.4	99.0	97.6	98.9
42	99.3	98.2	100.0	98.4	100.0

Supplementary Table S2. Percentile ranks norms for ES scores in the total sample (n = 1197).

Raw Score	Percentile Rank	95% Interval		90% Interval	
		Lower	Upper	Lower	Upper
4	1.2	0.0	2.7	0.1	2.5
5	3.2	2.0	4.8	2.1	4.5
6	5	3.6	7.4	3.8	7.1
7	8	5.9	9.9	6.1	9.6
8	11	8.2	13.1	8.5	12.7
9	14	11.2	16.6	11.6	16.2
10	17	14.4	20.3	14.8	19.9
11	21	18.0	24.3	18.4	23.8
12	25	21.7	28.2	22.2	27.7
13	29	25.6	32.7	26.1	32.2
14	34	30.1	37.8	30.6	37.2
15	39	35.0	42.4	35.5	41.9
16	44	39.7	48.6	40.3	48.0
17	51	46.2	55.4	46.8	54.8
18	57	53.0	61.7	53.6	61.1
19	64	59.4	68.6	60.0	68.0
20	71	66.4	74.9	67.0	74.4
21	76	72.7	79.8	73.2	79.4
22	82	77.8	85.5	78.4	85.1
23	87	83.8	89.7	84.2	89.3
24	91	88.1	93.4	88.5	93.1
25	94	92.1	95.7	92.4	95.5
26	96.3	94.5	97.8	94.8	97.6
27	97.8	96.7	98.6	96.9	98.5
28	99.1	97.8	100.0	98.0	99.9

Supplementary Table S3. Percentile ranks norms for CR scores in the female sample (n = 897).

Raw Score	Percentile Rank	95% Interval		90% Interval	
		Lower	Upper	Lower	Upper
6	0.7	0.0	1.9	0.0	1.7
7	1.8	1.0	2.9	1.1	2.7
8	2.8	1.6	4.3	1.8	4.0
9	4.0	2.7	5.6	2.9	5.4
10	5	3.8	7.2	4.0	6.9
11	7	5.0	8.4	5.2	8.0
12	8	5.9	10.2	6.2	9.8
13	9	7.6	11.6	7.9	11.3
14	11	8.8	13.4	9.1	13.0
15	13	10.7	16.3	11.0	15.8
16	16	13.3	18.8	13.7	18.3
17	18	15.6	21.6	16.0	21.1
18	22	18.4	25.0	18.8	24.5
19	25	21.7	28.7	22.2	28.2
20	29	25.3	32.7	25.9	32.1
21	32	28.9	35.7	29.4	35.1
22	36	31.8	39.8	32.4	39.1
23	40	36.0	43.7	36.6	43.1
24	44	40.0	48.8	40.6	48.1
25	50	45.3	53.9	45.9	53.3
26	55	50.3	58.8	51.0	58.1
27	59	55.2	63.6	55.8	63.0
28	64	60.0	68.1	60.7	67.5
29	68	64.4	71.7	65.0	71.2
30	72	68.1	75.6	68.7	75.1
31	76	72.3	79.4	72.9	78.9
32	80	76.2	82.7	76.8	82.3
33	83	79.8	86.2	80.3	85.7
34	87	83.7	90.0	84.2	89.6
35	90	87.7	92.5	88.1	92.1
36	93	90.3	94.9	90.7	94.6
37	95	93.0	96.6	93.4	96.4
38	96.3	94.8	97.4	95.0	97.3
39	97.3	95.8	98.5	96.1	98.3
40	98.2	97.1	98.9	97.3	98.8
41	98.6	97.5	99.2	97.7	99.1
42	99.4	98.3	100.0	98.5	100.0

Supplementary Table S4. Percentile ranks norms for ES scores in the female sample (n = 897).

Raw Score	Percentile Rank	95% Interval		90% Interval	
		Lower	Upper	Lower	Upper
4	1.3	0.0	3.2	0.1	2.9
5	3.8	2.2	5.8	2.4	5.4
6	6	4.2	8.7	4.5	8.3
7	9	6.8	11.7	7.1	11.3
8	12	9.5	15.3	9.9	14.9
9	16	12.9	19.7	13.4	19.1
10	20	17.0	24.1	17.5	23.5
11	25	21.1	28.4	21.6	27.8
12	29	25.2	32.8	25.7	32.2
13	33	29.4	37.4	30.0	36.7
14	38	33.7	41.6	34.3	41.0
15	42	37.9	46.4	38.6	45.8
16	48	43.0	52.3	43.6	51.6
17	54	49.0	58.5	49.7	57.9
18	60	55.3	64.5	56.0	63.9
19	66	61.5	71.2	62.2	70.6
20	73	68.4	77.1	69.1	76.5
21	78	74.3	81.7	74.9	81.2
22	83	79.1	86.7	79.7	86.2
23	88	84.5	90.6	85.0	90.2
24	92	88.6	94.1	89.0	93.8
25	95	92.4	96.4	92.8	96.2
26	96.8	95.0	98.2	95.2	98.0
27	98.2	97.0	99.0	97.2	98.9
28	99.3	98.1	100.0	98.3	100.0

Supplementary Table S5. Percentile ranks norms for CR scores in the male sample (*n* = 276).

Raw Score	Percentile Rank	95% Interval		90% Interval	
		Lower	Upper	Lower	Upper
6	0.9	0.0	3.2	0.0	2.7
7	2.2	0.8	4.5	1.0	4.1
8	2.9	1.3	5.5	1.5	5.0
9	3.4	1.7	6.1	2.0	5.6
10	3.6	1.9	6.3	2.1	5.8
11	3.8	2.0	6.6	2.2	6.1
12	5	2.6	8.5	3.0	7.9
13	7	4.1	10.2	4.5	9.6
14	8	4.9	11.2	5.3	10.6
15	9	5.9	13.5	6.4	12.7
16	12	8.0	16.0	8.5	15.3
17	14	9.9	18.5	10.4	17.7
18	16	12.0	21.4	12.6	20.5
19	19	14.6	24.5	15.3	23.6
20	22	17.0	27.1	17.7	26.2
21	25	19.4	30.4	20.2	29.5
22	28	22.8	34.2	23.6	33.3
23	33	26.5	39.1	27.5	38.0
24	37	30.9	42.9	31.8	41.9
25	40	34.3	46.8	35.2	45.7
26	45	38.2	51.0	39.2	50.0
27	49	42.4	55.1	43.4	54.1
28	53	46.7	60.1	47.8	59.0
29	59	52.4	66.2	53.5	65.2
30	66	58.9	71.9	59.9	71.0
31	71	64.7	77.2	65.8	76.3
32	76	70.2	81.7	71.2	80.9
33	81	75.1	85.8	76.1	85.0
34	84	79.0	88.0	79.8	87.4
35	86	81.3	90.0	82.1	89.4
36	88	84.0	92.0	84.7	91.5
37	91	86.4	93.9	87.1	93.4
38	93	88.8	95.4	89.4	95.0
39	95	91.2	97.3	91.8	97.0
40	96.7	93.9	98.6	94.4	98.3
41	97.8	95.5	99.2	95.9	99.0
42	99.1	96.8	100.0	97.3	100.0

Supplementary Table S6. Percentile ranks norms for ES scores in the male sample (n = 276).

Raw Score	Percentile Rank	95% Interval		90% Interval	
		Lower	Upper	Lower	Upper
4	0.7	0.0	2.8	0.0	2.4
5	1.6	0.6	3.7	0.7	3.3
6	2.4	0.9	4.9	1.1	4.4
7	3.4	1.6	6.3	1.9	5.7
8	5	2.6	8.5	3.0	7.9
9	7	4.0	10.0	4.4	9.4
10	8	4.9	11.9	5.4	11.2
11	10	6.8	14.4	7.2	13.7
12	13	8.7	17.1	9.3	16.3
13	16	11.4	21.7	12.1	20.8
14	22	16.1	29.1	17.0	28.1
15	28	22.4	33.9	23.3	32.9
16	34	26.7	40.8	27.7	39.6
17	41	34.1	48.8	35.1	47.6
18	49	41.7	55.8	42.7	54.7
19	56	48.8	63.8	49.9	62.7
20	64	57.0	71.0	58.1	69.9
21	71	64.1	76.7	65.1	75.8
22	78	70.6	84.1	71.7	83.2
23	84	78.7	88.9	79.6	88.3
24	89	83.9	93.3	84.8	92.8
25	93	88.9	95.6	89.6	95.2
26	95.1	91.6	97.6	92.2	97.3
27	96.7	94.0	98.4	94.5	98.2
28	98.6	95.7	100.0	96.2	99.9

The Polish version of the Emotion Regulation Questionnaire (ERQ)

Kwestionariusz regulacji emocji
Oryginalna wersja angielska: Gross i John (2003)
Wersja polska: Larionow i in. (2025)

Instrukcja i twierdzenia

Chcielibyśmy zadać Ci kilka pytań dotyczących Twojego życia emocjonalnego, w szczególności tego, jak kontrolujesz emocje (to znaczy jak je regulujesz lub jak nimi zarządzasz). Poniższe pytania obejmują dwa aspekty Twojego życia emocjonalnego. Pierwszy aspekt to Twoje <u>doświadczenia emocjonalne</u>, czyli to, co czujesz w środku. Drugi aspekt dotyczy Twojego <u>wyrażania emocji</u>, czyli tego, jak pokazujesz swoje emocje w sposobie mówienia, gestykulacji i zachowania. Chociaż niektóre z poniższych pytań mogą wydawać się podobne do siebie, różnią się one w istotny sposób. Odpowiedz, proszę, na każde pytanie, wpisując przy każdym twierdzeniu odpowiednią cyfrę z następującej skali:

**1**	**2**	**3**	**4**	**5**	**6**	**7**
**zdecydowanie się <u>nie zgadzam</u>**	--------------------	--------------------	**ani się zgadzam, ani się nie zgadzam**	--------------------	--------------------	**zdecydowanie się <u>zgadzam</u>**

1. ——— Kiedy chcę poczuć więcej *pozytywnych* emocji (takich jak radość lub rozbawienie), *zmieniam to, o czym myślę*.

2. ——— Trzymam swoje emocje w sobie.

3. ——— Kiedy chcę poczuć mniej *negatywnych* emocji (takich jak smutek lub złość), *zmieniam to, o czym myślę*.

4. ——— Kiedy odczuwam *pozytywne* emocje, staram się ich nie okazywać.

5. ——— Kiedy mam do czynienia ze stresującą sytuacją, staram się *myśleć o niej* w sposób, który pomaga mi zachować spokój.

6. ——— Kontroluję swoje emocje poprzez *niewyrażanie/nieokazywanie ich*.

7. ——— Kiedy chcę poczuć więcej *pozytywnych* emocji, *zmieniam swój sposób myślenia* o danej sytuacji.

8. ——— Kontroluję swoje emocje, *zmieniając swój sposób myślenia* o sytuacji, w której się znajduję.

9. ——— Kiedy odczuwam *negatywne* emocje, staram się ich nie okazywać.

10. ——— Kiedy chcę poczuć mniej *negatywnych* emocji, *zmieniam swój sposób myślenia* o danej sytuacji.

Instrukcja obliczania wyników

Kwestionariusz regulacji emocji (Emotion Regulation Questionnaire; ERQ; Gross i John, 2003) służy do oceny różnic indywidualnych w nawykowym stosowaniu dwóch strategii regulacji emocji: *przeformułowania poznawczego* (tj. zmiany sposobu myślenia o sytuacji w celu zmiany jej wpływu emocjonalnego) i *tłumienia ekspresji* (tj. tłumienia behawioralnej ekspresji emocji).

Wysoki poziom korzystania z przeformułowania poznawczego jest zwykle związany z dobrym samopoczuciem i wysoką jakością relacji interpersonalnych, podczas gdy wysoki poziom korzystania z tłumienia ekspresji jest zwykle związany z obniżonym samopoczuciem i niską jakością relacji interpersonalnych (Gross i John, 2003). Wyniki ERQ oblicza się oddzielnie dla każdej z tych strategii, przy czym wyższe wyniki wskazują na wyższy poziom wykorzystywania tych strategii.

Adnotacja

Nie zmieniaj kolejności pozycji, ponieważ pozycje 1 i 3 na początku kwestionariusza definiują terminy „pozytywne emocje" i „negatywne emocje".

Obliczanie wyników (brak pozycji odwróconych)

Przeformułowanie poznawcze: zsumuj wyniki pozycji 1, 3, 5, 7, 8, 10.

Tłumienie ekspresji: zsumuj wyniki pozycji 2, 4, 6, 9.
